# The Presidential Address before the American Institute, 1882

**Published:** 1882-11

**Authors:** Ad. Lippe

**Affiliations:** Philadelphia


### ADDRESS

### Delivered by the President of the American Institute, Indianapolis, June 13th, 1882.

This address, by order of the Institute, was printed in pamphlet form, and five thousand copies were distributed; it is now public property and open to criticism.

At the thirty-fifth session of this Institute, the President delivered an address in utter disharmony with the sentiments held and proclaimed by the founders of the Institute; also by the founder of the homœopathic healing-art. It is fortunately the first, and we hope the last, document of the kind, which must fill the hearts of the surviving founders and early members of the Institute with deep grief and mortification; were we to pass by this public document without criticism and comment the whole medical profession might reasonably infer, from such a negative indorsement, that the homœopathic healing-art, as promulgated by its founder and accepted by the founders of the Institute, had been found inadequate, found to be an illusion and a snare, and was wholly abandoned by the present members of the Institute and of the profession. That these erroneous impressions may be eliminated from the history of our healing-art we now undertake to show what a miserable address this is; that misrepresentations and false statements abound; that logic has been abandoned; and, finally, to show that the Institute is invited to resort to detestable means to finally wipe out the homœopathic healing-art and deliver the school over into the keeping of the most irrational, the most degraded of all schools of medicine that ever disgraced the medical profession, *the eclectic school of quackery.*

On page 3, we find the first logical blunder: "*The people cared less for the philosophy of Hahnemann's particular method than for practical results, and, believing success to be the test of merit, they have continued to encourage it,*" etc. Success could and can only be obtained if "the philosophy of Hahnemann's particular method" is fully accepted, and on that foundation only can we rest; a setting aside, caring nothing for Hahnemann's inductive methods (his philosophy), and falling into such errors as the speaker further advocates and recommends will leave us without that success the people expect to see. Furthermore, the orator's statement is *incorrect.* In the early days of Homœopathy in this country, practical results could not possibly be obtained before the correctness of Hahnemann's declarations of the successful cures made by his healing-art, based on his inductive methods, were fully established. The pioneers had to show that success, which became the test of merit, and so they addressed themselves to the intelligent portion of the community. Such pamphlets as Dr. Hering's *Rise and Progress of Homœopathy* awakened the people to hope for better results than they derived from the common school of medicine. Intelligent people then, as at present, sought to learn the philosophy of Hahnemann's methods of healing the sick. *After* becoming convinced that Hahnemann's methods, as explained in his *Organon of the Healing-Art,* were *theoretically* correct, they tried it by experiment, and, finding it true, ever after generously encouraged that system.

For the innovations made at later days when there could be an appeal made to the "success" of the pioneers, we can now well account. The philosophy of Hahnemann was no longer laid before the people, because many of the modern pretenders either did not know it, or, if they did, were too lazy to apply it consistently, and moreover, were not prepared to defend their increasing departures in the face of a well-read and well-instructed people.

Pages 4 and 5 read well, and inspire the unsophisticated reader with the false hope that the orator would defend Hahnemann's methods. Especially good and very happy are the quotations from the London *Lancet;* very applicable to the present condition of the Institute is the last sentence quoted from the *Lancet.* On page 6 the true inwardness of the orator is fully developed. He inadvertently utters one truth: "*Public opinion will not tolerate a base and transparent imitation.*"

Now the orator strikes his first blow to demolish our healing-art. To please "the regulars" we are admonished to be "*liberal,*" and while the regulars accuse us of practicing under an exclusive dogma, and for that reason decline to join us, we are told to teach them that while we believe the law of similars to be a general law, like the law of gravitation, we do not believe it to be an exclusive law in therapeutics! Here we are treated to logic with a vengeance. The law of gravitation is not only a general law but a universal law, or else it would be no law at all; so is the law of the similars either no law at all (a good method, Richard Hughes has it) or a universal law. The final illustration is worse than the illogical assertion. We are now boldly told that in absolutely incurable affections we claim that to the true physician the whole line of palliative treatment is open. A true healer, the true homœopathic physician, *never* resorts to the whole line of palliative* treatment. The true healer finds that, even in absolutely incurable diseases, the remedies applied under the universal law of the similars will, with positive and absolute certainty, better palliate and relieve the sufferings of the absolutely incurables, than will the "regular" and senseless administration of increasing doses of the ordinary palliatives; they not only do not satisfactorily relieve the sufferings, but invariably add new miseries to those already existing.

On page 8 a change comes over our learned orator when he says: "*Believing as we do in the stability of the law of similars, and its sufficiency in our guidance in medicinal therapeutics, we do not fear the result.*" "The stability of the law of the similars" must stand for universality, and what then becomes of the whole line of palliative treatment?

On page 9 the orator is guilty of plagiarism; there is no escape from the grave charge brought against him by Dr. Matthews.

On pages 10 and 11, the Public Health Association is credited with grappling with the "*inside sources of diseases.*" The learned orator might have given Hahnemann the credit due him, but he, the learned orator, probably never read the 5th paragraph, and the foot-note to paragraph 93 of Hahnemann's *Organon.*

On page 12 we are treated to a rehearsal of the great progress made toward disgracing our healing-art in the city of London, in 1881, at the International Homœopathic Medical Convention; that Convention we are now again told adopted for its standard: "*The broadest liberality of thought and freedom of medical opinion.*"

The most interesting part of the address is found in the considerations of the Institute's affairs. Page 16, the Committee on Publication are admonished to be more particular in examining the material that enters into the annual *Transactions.* It is to be regretted that the Committee of Publication allowed a succession of absurd fables to be published in the historical part (II) of the *Transactions* of the "World's Homœopathic Convention," held in Philadelphia in 1876. It must be a source of mortification to honorable men, that the Institute took not the slightest notice of the frequently published facts about the falsifications to be found in that volume. Whether the Committee of Publication will see to it that in future no more such

* Hahnemann's *Organon*, paragraph 67, and foot-notes.

transgressions are allowed, or whether they will only use their discretion to exclude papers written by members of the Institute who do not believe in the broadest liberality of thought and freedom of action, if *not* in harmony with the strict tenets of our healing-art, time alone will show.

The Bureau of Materia Medica is to be instructed to revise and *condense* our pathogenesy in hot haste, this boiled down product to be published by the Institute, after or while hastily boiling down our pathogenesy ; the same bureau is also expected to furnish a plan for the more *thorough* proving of drugs!

A Bureau of Pharmacy is proposed; a president of that bureau sitting in "star chamber", is to try and judge all homœopathic pharmacists, the bottle-washers, and other absurd vagaries, deprive them of all liberty of thought and freedom of action. Homœopathy in its struggle for scientific recognition can no better prevent scientific progress and scientific recognition than by establishing a "star chamber," to sit with closed doors and publish their findings in the *Transactions*, make a record of the pharmacists who (as the orator says) cannot escape much longer to be deprived of liberty of opinion.

On page 7 the learned President ventilates *his* peculiar joy over an even attempted "recognition" by the regulars, and he snaps at the American Medical Association, and expresses his ardent hope that "the Medical Society of the State of New York will assert its independence" and carry out their cunning design to wipe out Homœopathy as a distinctive school in New York, at least, just as they wiped out Thompsonianism almost half a century ago by their deceitful toleration. If the specialists in New York city read this address they may for a second time be lured into the erroneous belief that Homœopathy, as promulgated by the founder of the new healing-art, has been consigned to the tombs of the Capulets and is no more. Poor, deceived brethren of the medical profession! They will speedily find out that the sentiments uttered in the hope of a speedy recognition are not held by respectable, honest and consistent members of the homœopathic healing-art, who hold that the American Medical Association acted consistently, and could not have acted differently under the circumstances.

On page 20 the President becomes grandiloquent when he says: "*As a chief hindrance to the general and candid considerations of the truths of Homœopathy is the absurd doctrine, never taught by Hahne-*

*mann, of infinite dilution. We should endeavor to adopt some standard of limit for drug attenuation.*" This sentence is so absurd that it hardly merits criticism. Where is your boasted "broadest liberality of thought and freedom of medical opinion"? Where is it? To be confined to "star chamber" decisions? The chief hindrance to the general consideration of the truths of Homœopathy, are not the infinite dilutions; it is the distrust the profession at large has been taught to foster as to whether any truth can be found in Homœopathy when its practitioners violate all and every tenet of the school as promulgated by its founder, when they find the President of the Institute advocates palliative treatment, when they find him asking for recognition, and when they find him offering, as the price for that boon, to abandon Homœopathy as an exclusive system of therapeutics, founded on an eternal, positive and exclusive law of the similars. Has any scientific man of our days ever uttered such an absurdity as to recommend the adoption of a standard or limit for drug attenuation? All scientists of modern days, and among them medical teachers in allopathic universities (St. Petersburg, Stuttgart, etc.), have demonstrated "*scientifically*" the divisibility of matter and the most powerful and by division augmented effect of such divided matter on the human organism. Science assists us, but the President of the American Institute fables about the Institute's duty "*to adopt some standard or limit for drug attenuation!*" A limit, to be sure. And that is "science," is it? That is freedom of opinion, is it? And his silly proposition which must bring on the school the scorn and ridicule of all scientific men, is bolstered up by an absolutely erroneous statement, "*that ninety-nine out of every hundred homœopathic practitioners rely upon triturations and dilutions within the range ending at the tenth centesimal.*" We say this statement is "erroneous," and the orator knows it! And if it were correct so much more disgraced do these "ninety-nine out of every hundred" stand before the world! Disgraced, I say; and let the orator be informed that the school which he now tries to disgrace was first established in this country by men who gained for it recognition under the law of the land, and reputation among the intelligent people when ninety-nine out of every hundred at least cured *all* their cases with the 30th potency—not the 10th, which nobody then used. That is the true history, and the surviving old battle-scored pioneers know it. If the present ninety-nine, who see no curative power beyond the 10th, vote early and often for the limitation of drug action, that will neither alter the past history nor undo the thousands of cures daily made by such higher potencies as the practitioners feel the right and the liberty to administer. Another advocate of limitation says the 16th, another says the 14th, the President comes down to the 10th, while an Ohio philosopher says there is a limit at the 6th. Where is the argument, where are the proofs? We repeat, what we have often said before, that the testimony of one credible man who states that he cures the sick better, quicker and easier with a potency not used by the ninety and nine, is of much more value than all the efforts of the ninety and nine to deny facts of which they are not capable of judging, because of their inability to produce them. Again, as to this limitation suggestion: This question has been settled before, and the learned President is supposed to know something of the history of our healing-art. The documentary evidence contradicting all the bosh uttered lately, is to be found in the fourth volume of the *Œsterreichische Zeitschrift*, "Natrum muriaticum," by Dr. Watzke. And on page 251 will be found that ever-memorable confession of Dr. Watzke, the honest investigator, who says that unfortunately he finds himself compelled by the results of the experiments to confess that, contrary to the prevailing views, the 30th potency of kitchen salt has caused more symptoms, and, in its therapeutic application, has cured more sick, than the lower attenuations. *These* investigators were honest men. This was in 1848, and now, in 1882, the President of the largest body of "homœopaths" fables about a limitation to the 10th potency! Why? To please the common school of medicine for the sake of "*recognition!*" If that is progress in science let us ask for a halt just here and now.

The chief hindrance to the general and candid consideration of the truth of Homœopathy is not the posological question. The supposition that it is, implies that on *that question* the schools mainly differ. The differences were and are now the erroneous opinion held by the dominant school: that the same laws govern both inorganic bodies and organic bodies; that diseases have a material origin, and are, therefore, curable by material means, all of which the homœopathic healing-art completely reverses. Allopathic physicians who have adopted Homœopathy from "conviction" accepted these teachings and left, as every liberal healer will, the posological question open, to be settled in each *individual case* by the healer himself.

This address was delivered at the opening of the thirty-fifth session of the Institute. There were utterly ignored the teachings of the founders of the school, the labors and the fruits of the labors of the pioneers now no longer among us. Where is the memory of Gross, Stapf, Bœnninghausen, Wesselhœft, Hering, and last, but not least, our own Dunham, who never did suspect or dream that vipers he warmed and fed and brought back to life, the homeless and shelterless eclectics, are now stinging the hand that caressed them? When he so generously did plead for freedom in their behalf twelve years ago, he did not see their insincerity; *he*, so honest and true to the cause himself, could never suspect their final attempt to ask for recognition and their readiness for a complete surrender of principles for the sake of recognition.

Disgraceful as was the opening of this memorable thirty-fifth session, the close of it was by far more so. It was not only the privilege, but it was the solemn duty of the presiding officer to quelch the last resolution, offered while the session was closing, because it was "*unconstitutional.*"

We suggest that some of our notoriously eminent microscopists, notably, J. Edwards Smith, S. A. Jones and C. Wesselhœft, put this vile address and the last resolution offered and passed, by only one dissenting vote, at the close of the last session of the Institute, under their best instruments, and report whether they find the slightest trace of Hahnemann's healing-art or methods in either of them. All traces being lost, they might favor us still more if they can find falsifications, perversions, bad logic, or treason in them, and report to the Institute. AD. LIPPE.

*Philadelphia, September 15th,* 1882.

